# Supplementary figures and images for: Human coronaviruses disassemble processing bodies
Source: PLoS Pathog. 2022 Aug 23;18(8):e1010724. doi: 10.1371/journal.ppat.1010724 (PMC9439236; doi:10.1371/journal.ppat.1010724)

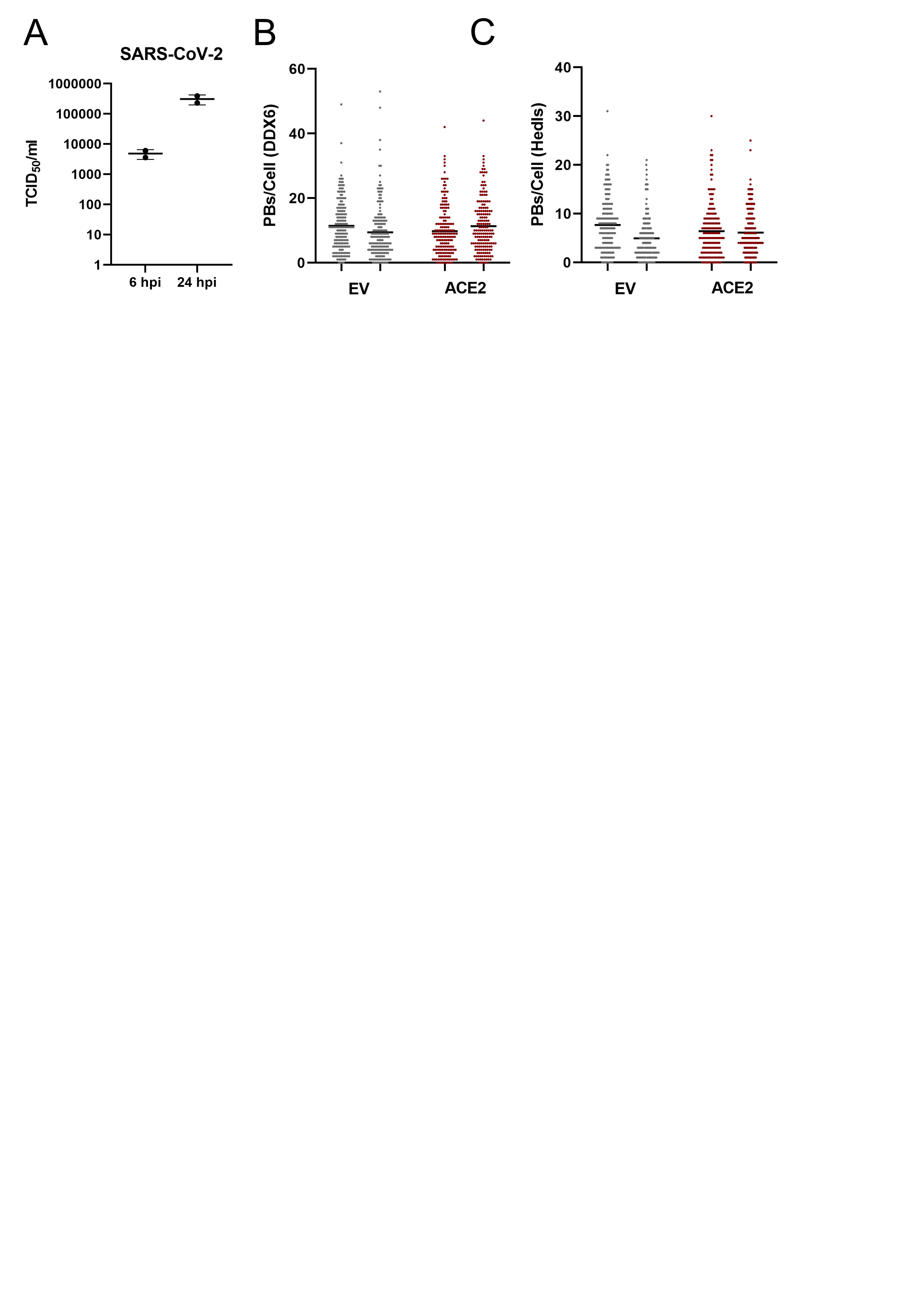

Supplement: S1 Fig — A. HUVECs were transduced with recombinant lentiviruses expressing human ACE2 (HUVECACE2), selected, and infected with SARS-CoV-2 (TO-1 isolate; MOI = 3). 6 and 24 hours post-infection, virus-containing supernatant was harvested and titrated by TCID50 assay in VeroE6 cells. These data represent two independent experiments (n = 2); mean ± SD. B-C. HUVECs were transduced with ACE2 as in A. Cells were fixed and immunostained for DDX6 (B) or Hedls (C) and quantified per field of view using CellProfiler. These data represent two independent experiments (n = 2) with >100 cells measured per condition. Each EV and ACE2 replicate pair plotted independently; mean. Statistics were performed using a Mann-Whitney rank-sum test (ns, nonsignificant). (TIFF) [file ppat.1010724.s001.tiff]

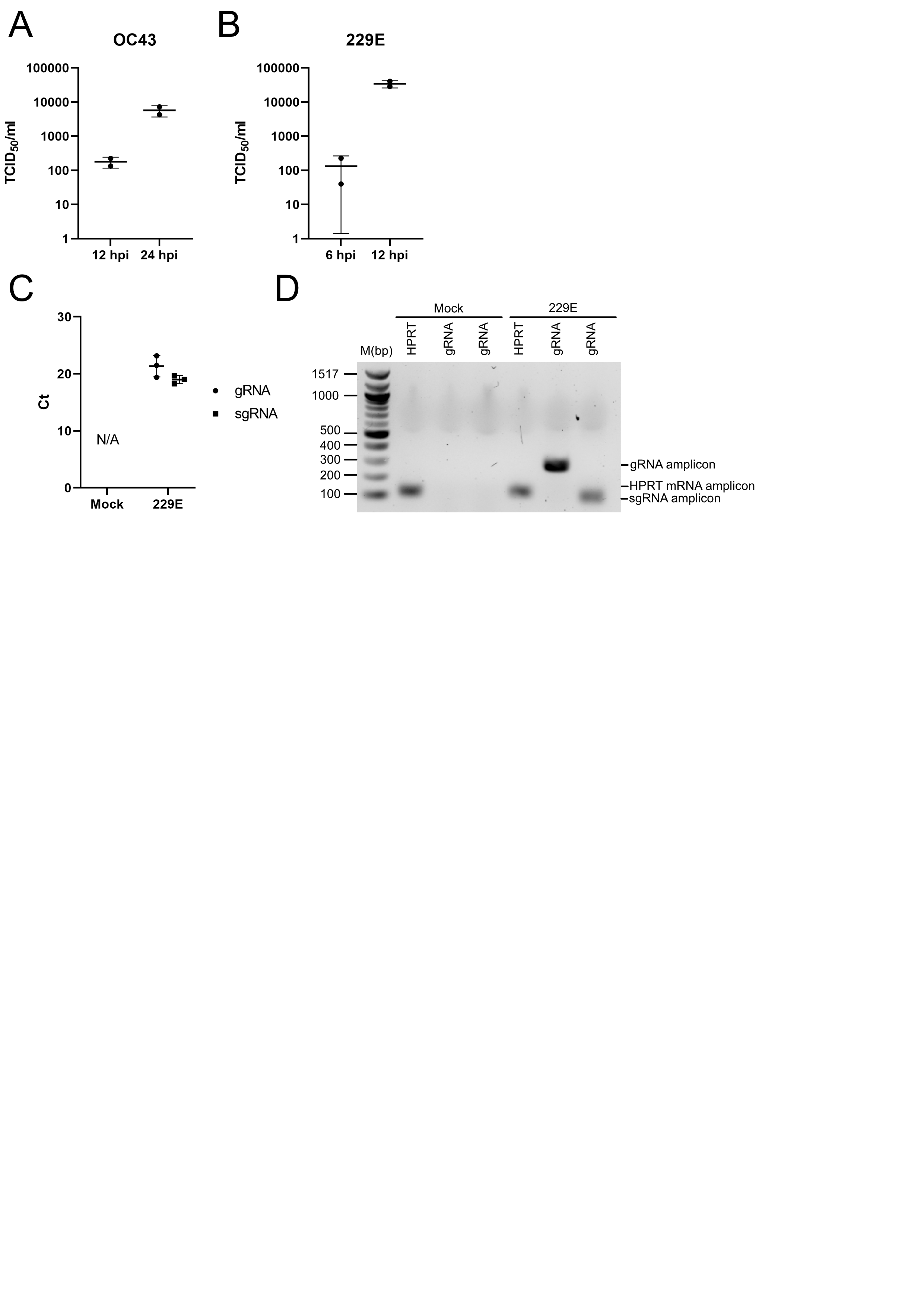

Supplement: S2 Fig — A-B. HUVECs were infected with OC43 (TCID50 = 2 x 10^4^) or 229E (TCID50 = 2.4 x 10^3^). 6-, 12-, or 24 hours post infection, virus-containing supernatant was harvested and titrated using TCID50 assay in VeroE6 (A) or MRC5 (B) cells, respectively. These data represent two independent experiments (n = 2); mean ± SD. C. HUVECs were infected with 229E as in B or mock infected. Total RNA was harvested 12 hours post infection and RT-qPCR was performed using primers specific to 229E genomic RNA (gRNA) or subgenomic RNA8 (sgRNA8), or HPRT (cellular house-keeping gene). Cycle-threshold (Ct) values for each primer pair were plotted. These data represent three independent experiments (n = 3); mean ± SD. Ct values greater than 35 were not deemed biologically relevant and therefore are scored as N/A. D. PCR products from C were resolved using agarose gel electrophoresis. gRNA fragment migrated at the expected size of 285 bp. sgRNA fragment migrated at its expected ~80 bp. Representative images from one of two independent experiments are shown. (TIFF) [file ppat.1010724.s002.tiff]

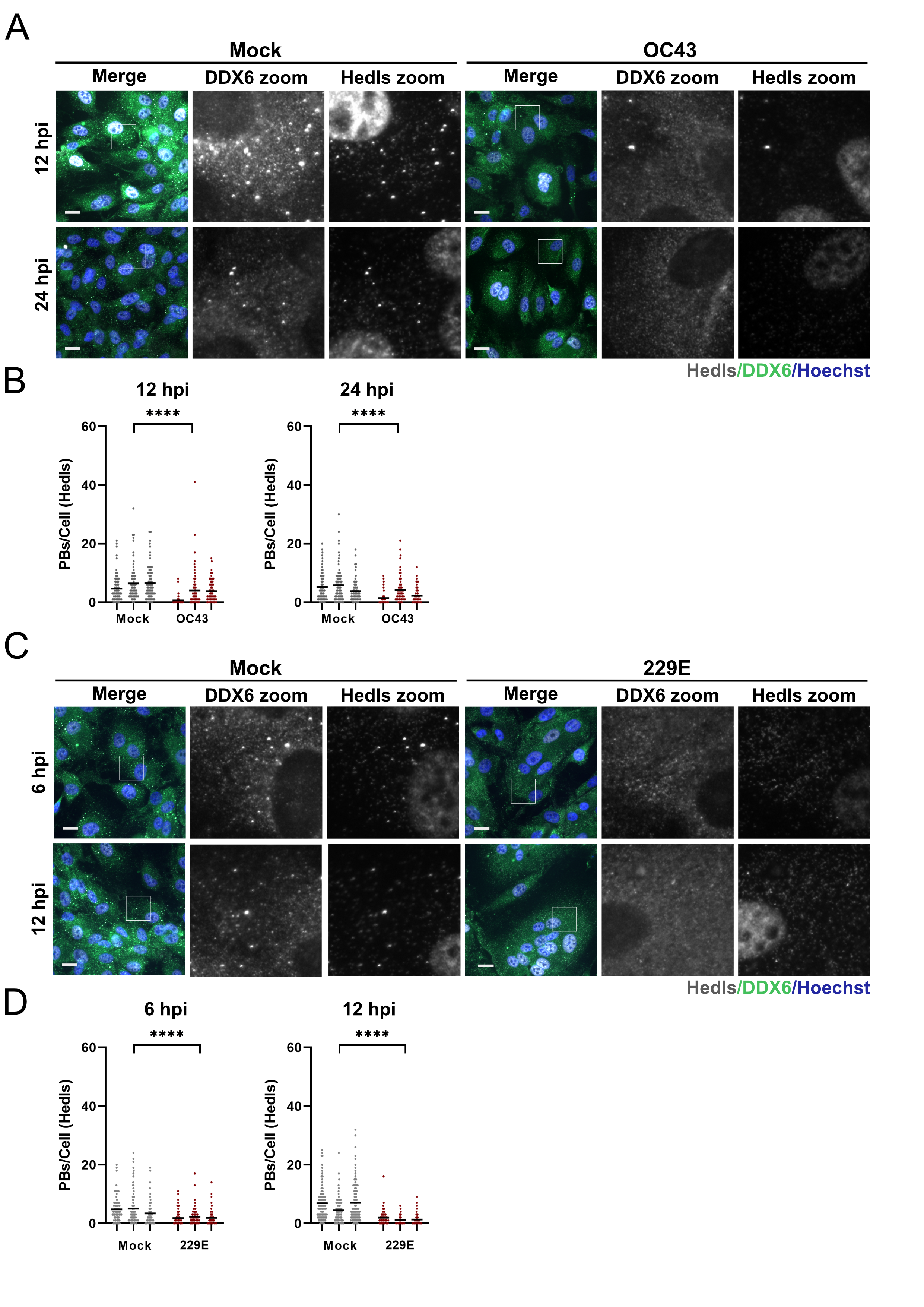

Supplement: S3 Fig — A. HUVECs were infected with OC43 (TCID50 = 2 x 10^4^) or mock-infected. 12 or 24 hours post infection cells were fixed and immunostained for DDX6 (green; Alexa488) and Hedls (white; Alexa555). Nuclei were stained with Hoechst (blue). Representative images from one of three independent experiments shown. B. Hedls puncta in OC43-infected or mock-infected cells were quantified per field of view using CellProfiler. C. HUVECs were infected with 229E (TCID50 = 2.4 x 10^3^) or mock-infected. 6 or 12 hours post infection cells were fixed and immunostained for DDX6 (green) and Hedls (white). Nuclei were stained with Hoechst (blue). Representative images from one of three independent experiments shown. D. Hedls puncta in 229E-infected or mock-infected cells were quantified as in B. These data represent three independent experiments (n = 3) with ≥100 cells measured per condition per replicate. Each mock and infected replicate pair plotted independently; mean. Statistics were performed using a Mann-Whitney rank-sum test (****, p < 0.0001). Scale bar = 20 μm. (TIFF) [file ppat.1010724.s003.tiff]

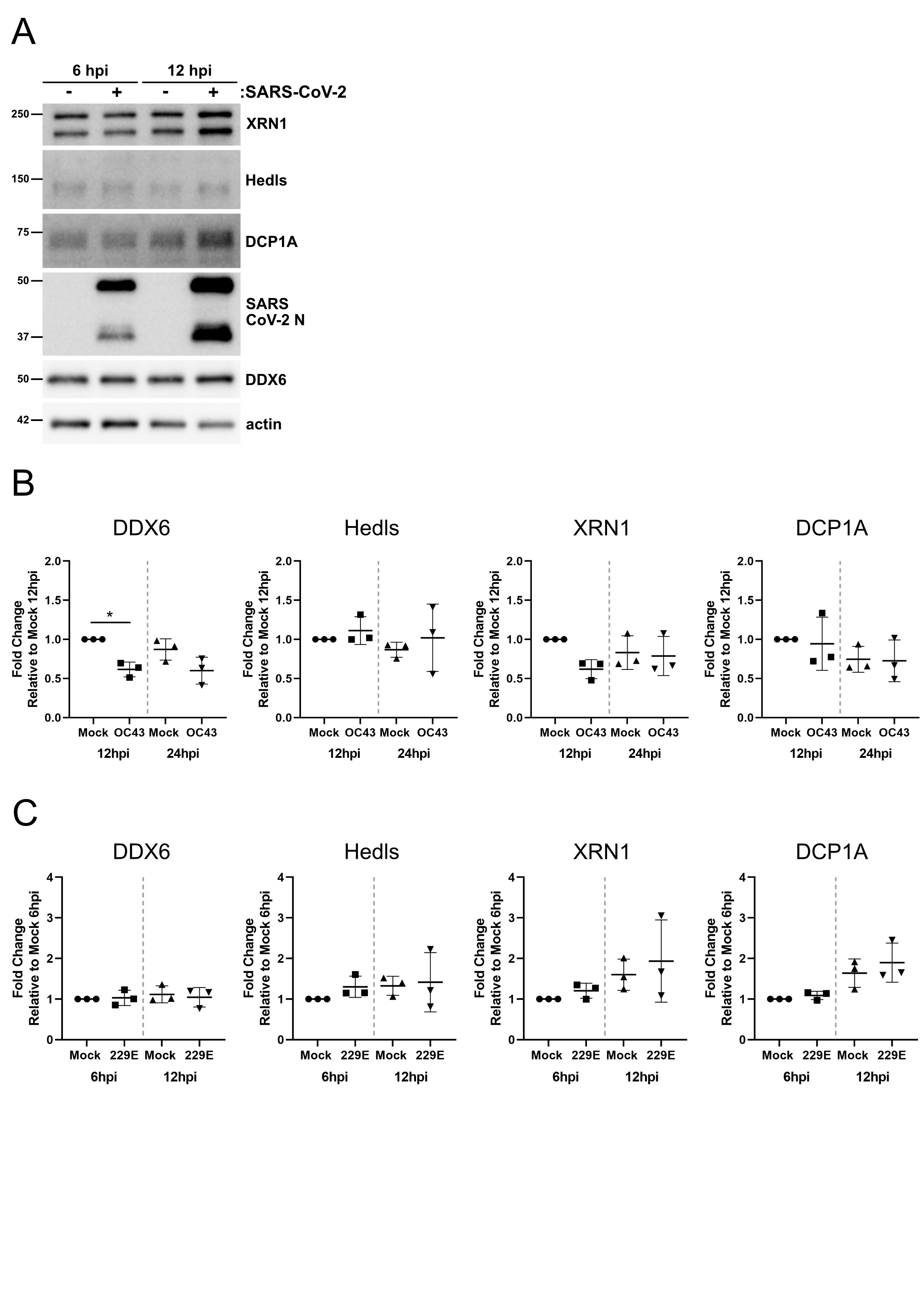

Supplement: S4 Fig — A. HUVECs were transduced with human ACE2 (HUVECACE2), selected, and infected with SARS-CoV-2 TO-1 isolate (MOI = 3). Cells were lysed at 6 and 12 hours post infection and immunoblotting was performed using XRN1, Hedls, DCP1A, DDX6, SARS-CoV-2 N, and β-actin specific antibodies. The results of the second independent experiment of two is shown. B-C. HUVECs were infected with OC43 (TCID50 = 2 x 10^4^) or 229E (TCID50 = 2.4 x 10^3^) or mock-infected. Cells were lysed at 12 and 24 hours post infection (B, OC43) or 6 and 12 hours post infection (C, 229E). Immunoblotting was performed using DDX6, Hedls, XRN1, DCP1A, and beta actin specific antibodies. Protein densitometry was determined in ImageJ normalized to beta actin and expressed as a fold-change relative to the 12 hours post infection (B; OC43) or 6 hours post infection (C; 229E) mock-infected control. These data represent three independent biological replicates (n = 3). A one-way ANOVA with a Dunnett’s post-hoc analysis was performed; mean; bars represent SD (*, p < 0.05). (TIFF) [file ppat.1010724.s004.tiff]

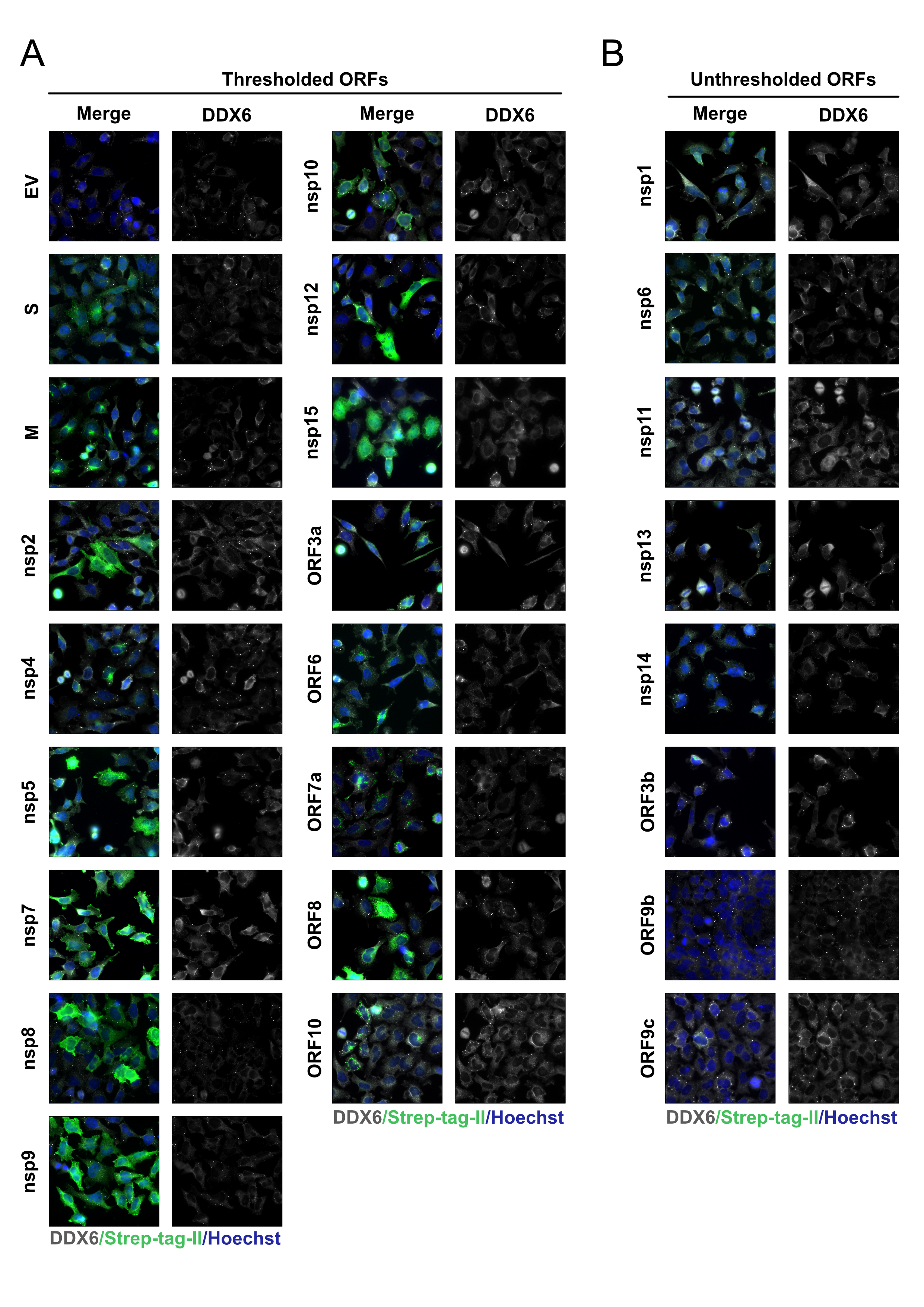

Supplement: S5 Fig — HeLa cells expressing GFP-Dcp1a were transfected with an empty vector (EV) control or 2X Strep-tagged SARS-CoV-2 ORFs for 48 hours then fixed and immunostained for Strep-tag II (viral ORF; green; Alexa647) or DDX6 (PBs; white; Alexa555). Nuclei were stained with Hoechst (blue). A. SARS-CoV-2 ORFs that could be detected by Strep-tag II staining (thresholded ORFs). B. SARS-CoV-2 ORFs that could not be detected by Strep-tag II staining (unthresholded ORFs). Representative images from one of three or more independent experiments shown. Scale is the same for all images. M = membrane protein, S = spike protein. (TIFF) [file ppat.1010724.s005.tiff]

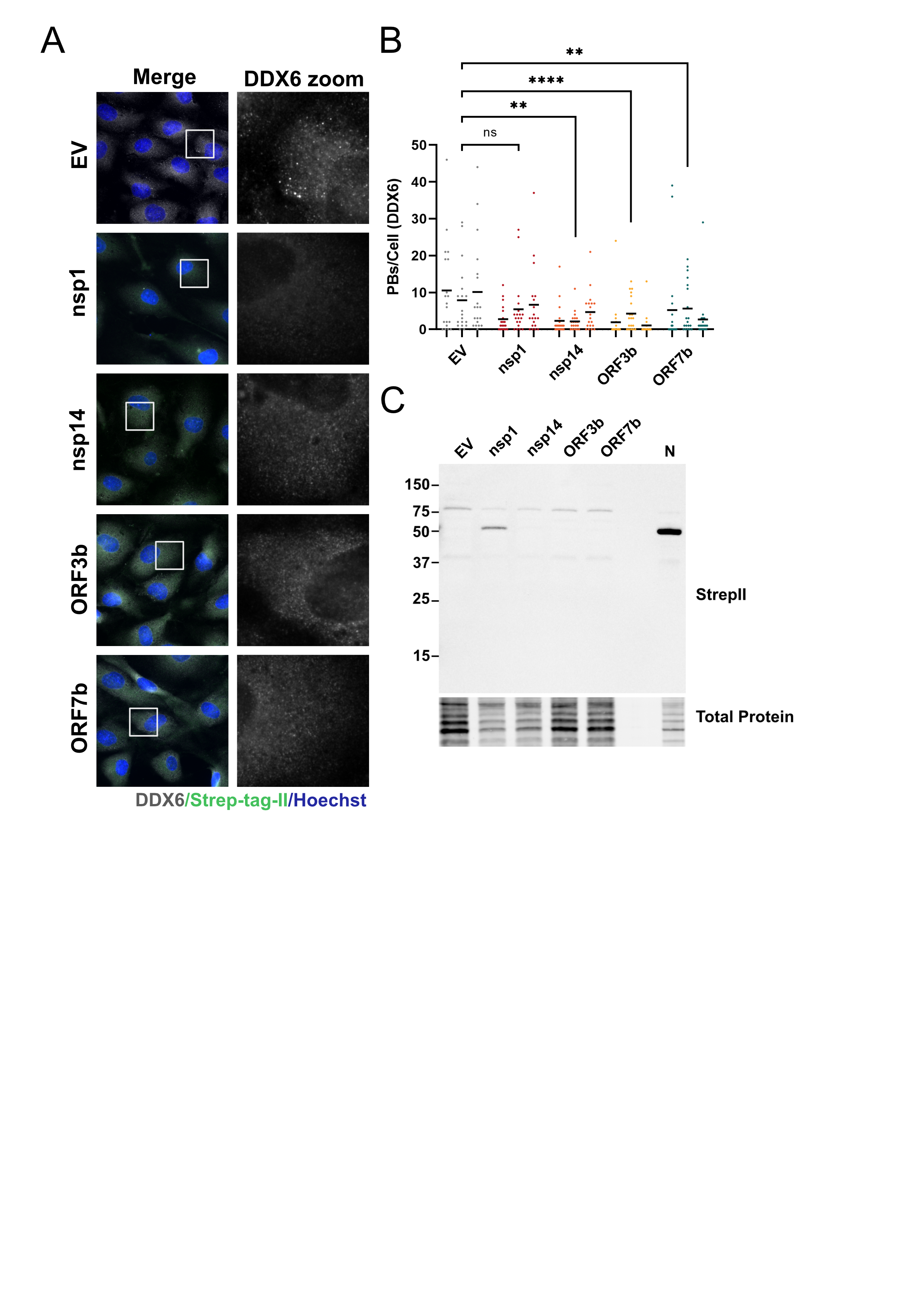

Supplement: S6 Fig — A. HUVECs were transduced with recombinant lentiviruses expressing 2X Strep-tagged SARS-CoV-2 nsp1, nsp14, ORF3b, and ORF7b constructs or control lentiviruses (EV) and selected with puromycin for 48 hours. Cells were fixed and immunostained for Strep-tag II (ORFs; green; Alexa488) and DDX6 (PBs; white; Alexa555). Nuclei were stained with Hoechst (blue). Representative images from one of three independent experiments shown. Scale bar = 20 μm. B. DDX6 puncta were quantified per field of view using CellProfiler. These data represent three independent experiments (n = 3) with >18 cells measured per condition per replicate. Each EV and ORF-expressing replicate pair plotted independently; mean. Statistics were performed using Kruskal-Wallis H test with Dunn’s correction (**, p < 0.0021; ****, p < 0.0001; ns, nonsignificant). C. HUVECs were transduced as in A. Cells were lysed and immunoblotting was performed using the Strep-Tag II antibody on a 4–15% gradient gel. (TIFF) [file ppat.1010724.s006.tiff]

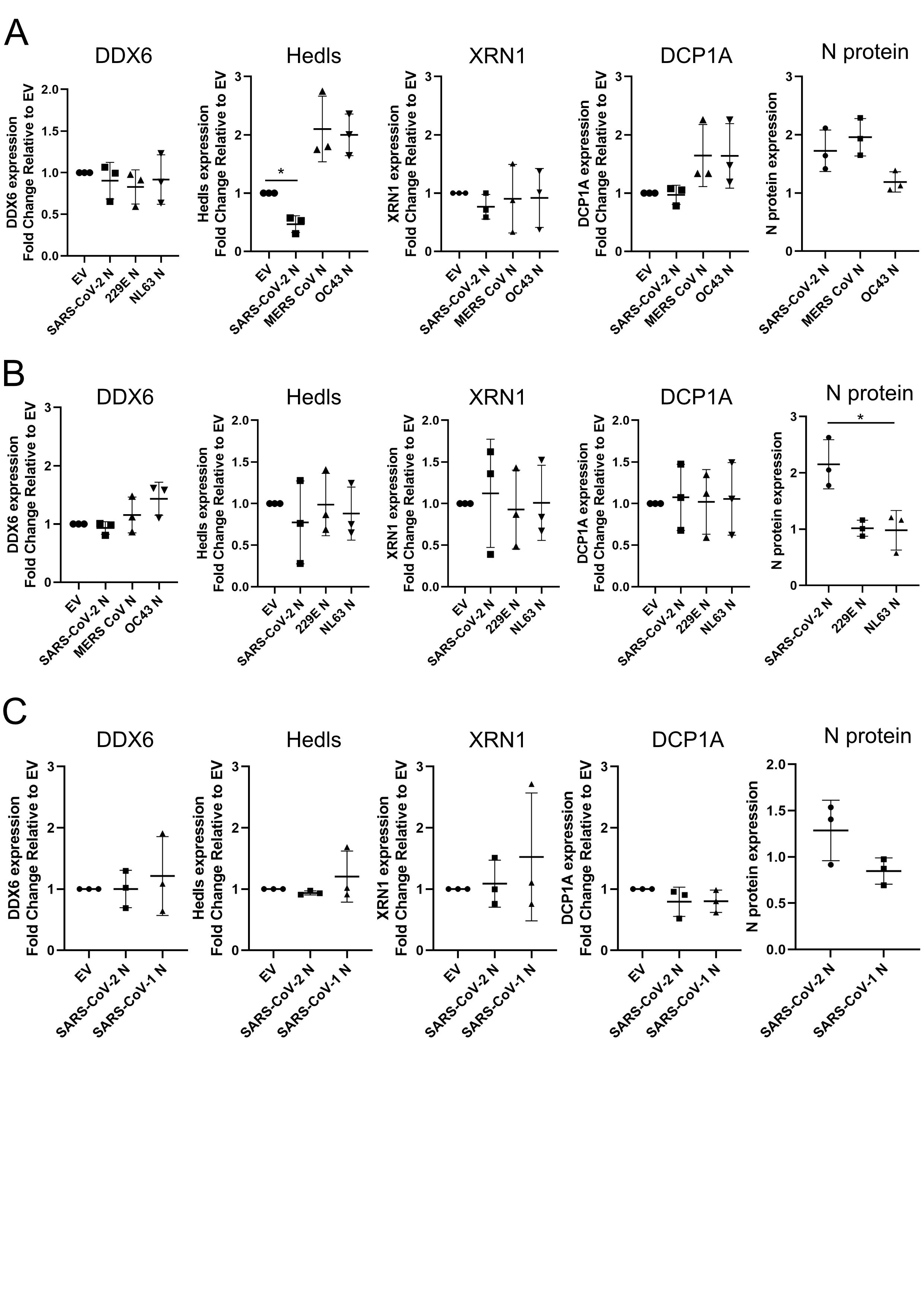

Supplement: S7 Fig — A-C. HUVECs were transduced with recombinant lentiviruses ectopically expressing N protein from the Betacoronaviruses MERS-CoV and OC43 (A), N protein from Alphacoronaviruses 229E and NL63 (B) or SARS-CoV-1 N (C). A control lentiviral expressing an empty vector (EV) was used as a negative control and SARS-CoV-2 N protein expressing lentiviruses were used as a positive control in each experiment. Cells were selected and lysed and immunoblotting was performed using XRN1, Hedls, DCP1A, DDX6, N protein or FLAG, and beta actin specific antibodies. Protein densitometry was determined in ImageJ normalized to beta actin and expressed as a fold-change relative to the EV control. These data represent three independent biological replicates (n = 3). A one-way ANOVA with a Dunnett’s post-hoc analysis was performed; mean; bars represent SD (*, p < 0.05). (TIFF) [file ppat.1010724.s007.tiff]

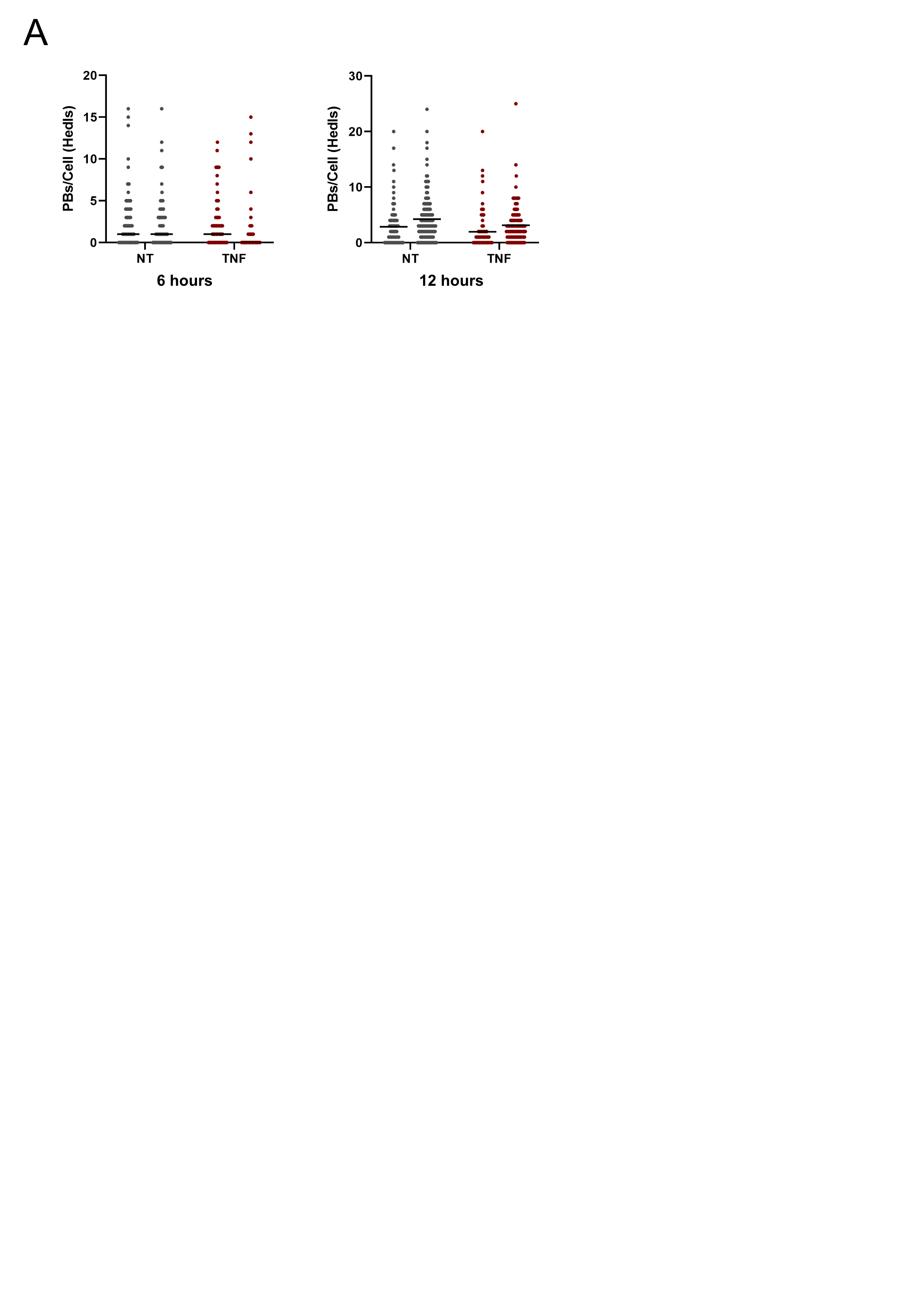

Supplement: S8 Fig — HUVECs were treated with 0.01 ng/L soluble TNF for either 6 or 12 hours following which, cells were fixed and immunostained for Hedls. Nuclei were stained with Hoechst. Hedls puncta were quantified per field of view using CellProfiler. These data represent two independent experiments (n = 2) with >90 cells measured per condition per replicate. Each mock and infected replicate pair plotted independently; mean. (TIFF) [file ppat.1010724.s008.tiff]

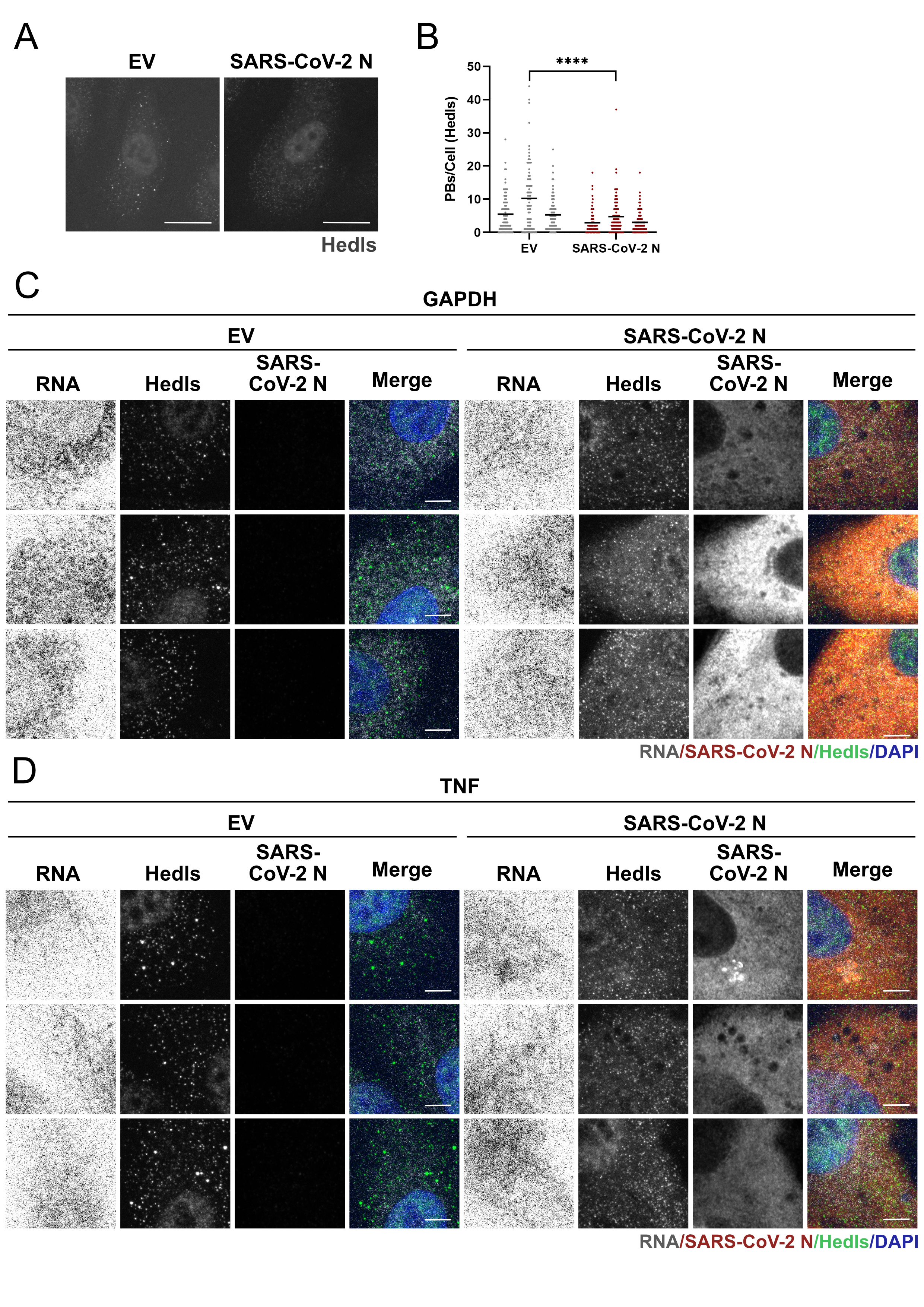

Supplement: S9 Fig — A-B. Cells were treated and immunostained as in Fig 9B and Hedls puncta (white; Alexa488) were quantified per field of view using CellProfiler. Scale bar = 20 μm. These data represent three independent experiments (n = 3) with >75 cells measured per condition per replicate; mean. Statistics were performed using a Mann-Whitney rank-sum test (****, p < 0.0001). C-D. HUVECs were transduced with recombinant lentiviruses ectopically expressing SARS-CoV-2 N or an EV control. Cells were selected, fixed and immunostained for either Hedls (green; Alexa488) and SARS-CoV-2 N (red; Alexa555) prior to hybridization with Stellaris probes specific for GAPDH and TNF (grey; Quasar 670). Nuclei were stained with DAPI. Cells were imaged using a Zeiss LSM 880 laser scanning confocal microscope and the 63X objective. Representative images from two independent experiments are shown. Each channel is shown separately in black and white, including an inverted image of the RNA FISH channel, prior to merged images. Scale bar = 5 μm. (TIFF) [file ppat.1010724.s009.tiff]
