# Supplementary material for: Human coronaviruses disassemble processing bodies
Source: PLoS Pathog. 2022 Aug 23;18(8):e1010724. doi: 10.1371/journal.ppat.1010724 (PMC9439236; doi:10.1371/journal.ppat.1010724)
Supplement: Attachment — Submitted filename: Corcoran Kleer Mulloy Response to Reviewers.pdf [file ppat.1010724.s010.pdf]

Reviewer #1: Major

1. The direct relation of virus induces PB loss and the elevated transcript levels of selected proinflammatory cytokines is poor. The authors should examine the levels of other mRNAs, such as growth factors and angiogenic factors.
The fact that all RNAs do not respond to changes in PB dynamics the same way is noted in the paper with strong reference support (Mol Cell paper) but not discussed at length. If we have RNA, why don't we look at growth factors or VEGF?

We thank the reviewer for their comments about our data. Given the current knowledge in the field, it remains difficult to reconcile why some AU-rich element (ARE)-containing RNAs respond differently to PB disassembly than others. The relationship between PB form and function is at the crux of the reviewer's comment, and these questions are ones that are being grappled with in the field at large. We do not precisely understand the functional significance of PB disassembly on cellular gene expression, and this is a question that is currently under investigation by several labs, including ours. A comprehensive analysis of the RNA and protein content of PBs (isolated granules by fluorescent particle sorting) was informative, as this study identified that certain groups of RNAs are preferentially found in PBs (those that contain AU-rich elements, those that encode potent regulatory molecules like cytokines, and those that are not highly translated) while other transcripts appeared to be preferentially excluded from PBs (highly translated RNAs and housekeeping transcripts) (Hubstenberger, 2017). The strongest evidence for elevation of a distinct group of RNA transcripts in response to PB disassembly derives from our lab and others, as we have shown that transcripts that bear 3'-UTR AREs often increase in response to PB disassembly (Corcoran, 2012, Corcoran 2015, Robinson 2022, Castle, 2021, Kleer 2021, Vindry 2017, Blanco 2014, Tenekeci 2016). However, all ARE-containing mRNAs (there are ~4500 cellular transcripts contain these elements, Bakheet 2018) do not respond the same way to PB disassembly, as others have shown and as we observe in this manuscript (Tenekeci 2016, Al-Souhibani 2010, Seto 2015). In our study, we focused on ARE-containing transcripts that were relevant to CoV infection, and we observed IL-6 and TNF RNA levels increased concomitant with PB disassembly, while levels of other RNAs we tested did not (Figs 5, 9, 10). Moreover, in new data added to the revised manuscript, we used RNA FISH to show that these transcripts are found in PBs (Fig 9). In response to reviewers' concerns, we added analysis of COX-2 to Fig 10 but did not observe a significant increase in COX-2 mRNA levels in the context of N protein expression (Fig 10D).

2. The mRNA in the PBs can be displayed in FISH assay, which is a direct evidence authors can prove that the mRNA of inflammation factors is imprisoned in PBs (if it is indeed inside).

We thank the reviewer for this insightful suggestion. We performed additional experiments to label AU-rich cytokine transcripts using fluorescent *in situ* hybridization (FISH) and added these data to the revised manuscript (Fig 9). We used Stellaris probes to perform RNA FISH for three cellular transcripts: GAPDH, which is not regulated by PBs and would not be expected to localize to PBs or respond to changes in PB dynamics, and IL-6 and TNF, which both contain AU-rich elements that direct them to PBs. Using IF-FISH, we now show that TNF and IL-6 RNA transcripts co-stain with PB foci (labeled with the antibody to Hedls) while GAPDH transcripts do not. Moreover, we show that in cells expressing the N protein, when PBs are

absent, total intensity of FISH staining for IL-6 and TNF transcripts is increased and the staining profile for these transcripts shows that they re-localize to the cytoplasm. Staining for GAPDH RNA remained largely unchanged (Fig 9). This suggests that the biological relevance of PB disassembly derives from two distinct consequences of disassembly: 1) the increased abundance of ARE-mRNA transcripts that are subjected to constitutive turnover in PBs and 2) the redistribution of these transcripts from PB foci to the cytoplasm where they can access cellular ribosomes for translation.

3. The cellular factor level of five types of ARE-containing was detected. Why did only three expression levels increase? If the author believes that the increase is because of the depolymerization of PBs, even if it does not detect other types of Are-containing RNA included in the PBs, at least these five should be expressed in similar level (figure 4)

PBs are sites of post-transcriptional regulation that dictate what mRNA transcripts are made into protein. Much remains unclear about how individual mRNAs are shuttled to PBs, and even less is known about why some transcripts increase in abundance upon PB disassembly while others do not. Certain classes of cellular transcripts, including those that contain AU-rich elements (AREs) in their 3'UTRs, are much more likely to be found in PBs (Hubstenberger, 2017). These typically encode molecules with potent regulatory roles which is why their translation into protein is so tightly regulated. As noted above, the PB field continues to grapple with this issue; to solve the problem of the field in this manuscript is well beyond the scope of the current study.

4. Inhibition of PBs might indeed release some mRNA, but I tend to think that these mRNAs are regulatory factors that promote transcription of those cytokines.

Our new data (Fig 9) showed that TNF and IL-6 cytokine mRNA transcripts localized to PBs under basal conditions, confirming that these are PB-regulated RNAs. Moreover, we showed that ectopic expression of N protein disassembles PBs and increased the overall RNA FISH signal for both these transcripts, while the signal for GAPDH RNA was not significantly elevated. These data support our model whereby cytokine transcripts constitutively shuttle to PBs where they are either housed and repressed or degraded until PB disassembly mediates their release.

5. If PBs play antiviral effect with the infection of the SARS-CoV-2, then will inhibition of the formation of PBs promote viral infection? Authors should clarify this point.

We thank the authors for this insight. We do believe that PB disassembly is an important feature of a successful viral infection. Numerous publications have shown that a breadth of viral families induce PB disassembly, strongly suggesting that PBs have an antiviral role. We therefore hypothesize that PB fortification is antiviral. Our companion hypothesis is that viral-mediated PB disassembly is a rapid switch that induces stabilization and redistribution of cytokine RNA transcripts to permit their enhanced translation, thereby alerting the immune system to the viral infection. Ongoing work in our laboratory is striving to understand the basis of this mechanism. This literature supporting an antiviral role of PBs is described in the introduction, while there are two paragraphs in the discussion of the revised manuscript on this point.

6. The results of immunofluorescence should not only show the picture of merge but also show the separate figure.

We apologize for our confusion; however, we are unclear what the reviewer is requesting. All immunofluorescence images show the PB channel separated from the merged image. If the reviewer means that each image should be separated out at lower magnification in addition to the zoom panel, we considered this option; however, this would cause a space issue in a paper that already has 10 main figures and 8 supplemental figures.

Reviewer #1 Minor Concerns

1 Figure 1D: authors should add the figure of cells infected with SARS-CoV-2 of 16h post-infection.

In Fig 1 of the revised manuscript, we have added additional images and quantification of PBs for 16 hours post infection with SARS-CoV-2. Moreover, we significantly strengthen our finding by performing infections with four prevalent SARS-CoV-2 variants of concern (VOCs) and show that Alpha, Beta, Gamma and Delta each induce PB disassembly (Fig 2 of revised manuscript).

2 Figure 1F: authors should show more time of Calu3 cells infected with SARS-CoV-2.

In Figure 1F, we have added additional analysis of SARS-CoV-2-infected Calu-3 cells at 24hpi and quantified PB loss at this time point. We have also stained infected Calu-3 cells for two different PB proteins, Hedls and DDX6.

3 Figure 2C and 2E: Most of cells (mock) show no PBs, thus, how can authors prove that OC-43 and 229E truly inhibit PBs.

It is true that some cells in our mock infected condition do not contain PBs. The number of PBs per cell is variable between experiments, especially when we use primary cells like HUVECs. To make this variability more transparent to the reviewer and the reader, we have re-graphed the PB counts from Fig 2 and all other figures to show the number of PBs per cell for every cell counted in every experiment. This increases the transparency of our data and will provide the reviewer with the confidence that the PB counts per cell are significantly different after infection with OC43 and 229E (Fig 2 from previous version, Fig 3 in revised version of the paper).

4 Figure 4: Why do authors only examine the level of mRNA. It is required to detect the level of proteins too.

We agree with the reviewer that the analysis of protein levels is an obvious next step in this study. However, the current revised version of this manuscript contains 10 main figures and 8 supplemental figures, so we deemed these analyses to be beyond the scope of the current work. In the introduction section, we have also referred to several studies that showed increases in IL-6 and TNF protein levels during SARS-CoV-2 infection.

5 Figure 5D: The expression of nsp4, orf9c, orf10 is not examined.

Expression of nsp4, ORF9c and ORF10 were not detected by immunoblotting (Fig 6D of the revised manuscript), but were detected by immunofluorescence, as now shown in a new supplemental figure (Fig S5). When we quantified PBs in cells expressing nsp4 and ORF10, we counted PBs only in cells that were visibly expressing the indicated construct. This is reflected in Fig 6B (thresholded ORFs for PB counts). Although expression of ORF9c was visualized by immunofluorescence, the low staining did not permit counting PBs in only transfected cells using our CellProfiler pipeline (Fig S5); for that reason, PB counting was unthresholded and PBs were counted in all cells (Fig 6E). We have added additional statements to clarify this in the results.

6 Figure 5: The Immunofluorescent results of other proteins of coronaviruses should be supplied.

We have included images of all 27 SARS-CoV-2 gene products tested in our screen – these data are shown in Fig S5 of the revised manuscript. As noted in the results, we were unable reliably detect which cells were expressing some of the constructs; therefore, analysis of PB counts after transfection of low-expressing gene products was performed in an unthresholded manner, and all cells in the monolayer were counted in the analysis.

7 Figure 6: result show that N protein cannot inhibit PBs induced by Ars. What is the difference between PBs of the normal situation and PBs induced by Ars?

Although PBs are constitutive ribonucleoprotein granules that are found in most cells, they are dynamic, always dissolving and reforming even under basal conditions. Moreover, different treatments or stressors have been shown to influence this homeostasis, thereby altering PB size and number. When a treatment or protein induces loss of PBs, it is important to differentiate between two possibilities – did the treatment enhance PB disassembly or prevent assembly? The purpose of treating N protein-expressing cells with sodium arsenite is to differentiate between these two possibilities, as we did in (Robinson 2022). Sodium arsenite (oxidative stress) activates the integrated stress response to shutdown cellular translation, the amount of cellular RNA increases, and this is presumed to be the reason for the increased formation of PBs under these conditions (Kedersha 2006). Because we observed that N protein expression did not prevent PB assembly after arsenite treatment, it is unlikely that N works by eliminating one of the essential factors required for PB formation (DDX6, 4E-T, Lsm14A) as noted in (Ayache 2015).
We concluded that N expression altered PB dynamics by inducing PB disassembly (Fig 7 in the revised manuscript).

8 Figure 7A and B: Why don't you use SARS's N protein? SARS's N should have a similar phenomenon.

We appreciate the point made by the reviewer. We initially had some difficulty subcloning SARS-CoV-1 N into our lentiviral transfer vector; however, this difficulty has been solved and we have now tested the ability of SARS-CoV-1 N to disassemble PBs in HUVECs. These new data have been added to Fig 8E-F.

Reviewer #2: Major

1. Positive controls. Throughout the introduction you indicate that PB disassembly occurs often during viral infection and in Lines 75-76 you state that: "the presence of PBs correlates with presence/suppression of ARE-mRNAs [7,12-15]". The addition of a positive control known to disassemble PBs (treatment of viral product) would strengthen the hypothesis that the two observations (loss of PBs and increased levels of cytokine transcripts) are linked. Especially because not all ARE-mRNAs tested display similarly elevated levels upon PB disassembly by coronaviruses or SARS-CoV-2 N specifically. If PB disassembly is the sole reason for the enhanced presence of IL-6, COX-2 and or TNF, should we not expect the same pattern when PBs are disassembled by other treatments?

We have previously published work on a Kaposi's sarcoma-associated herpesvirus (KSHV) protein called KapB that induced PB disassembly (Corcoran 2015, Castle 2021, Kleer 2021, Robinson 2022), increased the stability of an ARE-containing luciferase reporter gene (Corcoran 2015, Castle 2021, Robinson 2022) and increased mRNA levels for some ARE-containing cytokine transcripts (Robinson 2022). We had included KapB in our initial screen of SARS-CoV-2 genes as a measure of successful PB disassembly. We have added these data to the revised version of Fig 6B, C.

2. Experimental design is not clearly described. All figures: what is n? are these three independent experiments, three wells, of three 'field of views'? When fold change is presented (1CE, 2DF, 4, 5BC, 6BDF, 7BD, 8, S1A and S2B) it is not always clear what is depicted. Cells expressing PBs? Or PBs per cell? Figure 5BC describes puncta/cell, so how many cells were analyzed from how many wells in how many independent experiments?

We sincerely apologize to the reviewer for the lack of clarify regarding our figures/data presentation. We have amended each of the figure legends to be clearer. Moreover, we have re-graphed all our PB counts to represent PBs/cell for every cell counted in each experiment. Every figure that showed PB counts has been re-graphed in this manner, thereby providing greater data transparency for the reviewer.

3. Most figures are expressed as fold change (1CE, 2DF, 4, 5BC, 6BDF, 7BD, 8, S1A and S2B). In none of these figures the mock or empty vector that samples are normalized to contains error bars. This suggests that for each data point all samples were normalized to the mock, before the average of n=3 was calculated. If this is the case this removes all variance in the mock group and increases the probability that comparison with a sample population results in significant differences. If the authors insist on presenting fold change, the variance in the mock treatment should be maintained, SDs for mock and EV should be presented and statistics should be performed on the raw data.

We have re-graphed all our PB counts to represent PBs/cell for every cell counted in each independent experiment. Every figure that showed PB counts has been presented in this manner, thereby providing greater data transparency for the reviewer and reflecting the variability of PB counts in our mock or empty vector control conditions. Statistical analysis has been performed on raw data (PB counts) as denoted in more detail in the methods and figure legends. In addition,

we have re-analyzed all of our RT-qPCR analysis to depict variance in the mock group (new Fig 5 and Fig 10).

4. Immunofluorescent images are used to quantify PBs with a previously established pipeline. The materials and methods describes clearly that quantification with the cellprofiler pipeline was done using consistent thresholds and identical parameters (Lines: 543-544). In some images DDX6 or Hedls containing granules are clearly visible but they do not make the cutoff and are displayed as examples of PB negative cells (e.g. virus infected Zoom images of Figures 1 and 2). To make a fair comparison, not only subsequent quantification analysis but also initial exposure times have to be identical between PB positive and negative (mock and infected) samples. Please provide key information on the exposure times and additionally list the make and model of the microscope.

We thank the reviewer for their attentiveness to the nuances of our data. Exposure times are always equal between mock and infected/overexpression samples for all images to ensure PB thresholding and counts are accurate. We have added additional details to the methods to reflect this point more accurately. We have also included additional data in all figures by re-graphing our PB counts to represent every cell counted in each experiment.

5. Unclear why in figure 4 COX-2 is examined and found to change significantly upon SARS-CoV-2 infection, while after figure 4 the COX-2 transcript is not mentioned again and for figure 8 TNF is measured. Line 290 even states TNF was elevated after virus infection (Fig.4), while I cannot find TNF anywhere in figure 4. Without clear arguments for this inconsistency, using COX-2 in Fig.4 and TNF in Fig. 8 feels like cherry picking. Furthermore, please clarify why only for Fig. 8 and not Fig. 4 cells were stimulated with TNF.

We thank the reviewer for this point. Fig 4 (Fig 5 in the revised manuscript) shows RNA analysis after infection, while Fig 8 (Fig 10 in the revised manuscript), shows RNA analysis after ectopic expression of hu CoV N proteins. We did attempt to measure TNF transcript levels after infection, but RNA levels were too low (Ct values too high) in the uninfected control to be able to reliably measure any changes in RNA level under these circumstances. In the revised paper, we added COX-2 transcript analysis to the revised version of Fig 10, as requested by the reviewer. We used TNF to stimulate cytokine transcription in Fig 10 because these cells would not be expected to induce cytokine transcription under basal conditions. However, in Fig 5, we expected that viral infection would sufficiently stimulate transcription of ARE-containing cytokines for our analysis by RT-qPCR. Still, in the case of TNF RNA, we were unable to detect high enough levels of TNF in the uninfected control to make the analysis accurate.

Reviewer #2: Minor

Lines 45-47: Sentence unclear. The relevance only becomes clear after reading the introduction of the manuscript. Moreover, also line 55 describes transcript levels as 'repressed'. When reading the abstract on its own I am left wondering where? How? and why? Again this all becomes perfectly clear in the introduction of the main text.

We apologize for the unclear abstract. We have rewritten the abstract with the reviewer's concerns in mind.

Lines 57-60 and lines 108-111: The conclusion that proinflammatory cytokine mRNA levels are increased as an unintended side-effect of SARS-CoV-2 infection is a bit of an odd and counterintuitive note. Firstly, by calling it an unintended side effect it suggests there is no benefit for the virus, leaving no room for tradeoffs with potential positive effects on virus transmission in a natural situation. Secondly, refrain from using the word 'unintended' as viruses do not act with intent.

We thank the reviewer for pointing out our incorrect use of anthropomorphic language regarding viruses. We have amended the abstract to remove the word unintended.

Line 164: Could use a rationale why Fig. 1F is performed at 48 and not 24hpi

In our initial experiments, we infected Calu-3 with a lower MOI of SARS-CoV-2 and found PB disassembly was greater at 48 hours post infection. However, when we repeated these experiments for the revised version of the paper, we used a higher MOI and observed greater PB disassembly at 24 hours post infection. In Fig 1, we now show representative images and quantify PB disassembly Calu-3 cells at 24hpi.

Lines 193-194: Fig. 3C, was infection with 229E confirmed?

We do not have an antibody for 229E N protein; therefore, for immunofluorescence experiments, we have identified infected cells using the anti-dsRNA monoclonal antibody (J2). However, in response to the reviewer's concerns, we performed an additional experiment on 229E-infected cells and have analyzed viral RNA accumulation by RT-qPCR, as well as performed TCID50 to detect infectious viral progeny in the supernatant of 229E-infected HUVECs. These confirmatory experiments are presented in Fig S2 of the revised manuscript.

Figure 5B-D: Viral protein products are labelled differently (E vs env, S vs spike etc)
Figures: abbreviation EV (empty vector) should be explained well before the legend of Figure 8.

We have changed the labels in Fig 6 and Fig S5 of the revised paper to be consistent. We have defined these abbreviations in the new figure legends.

Figure legends
Line 548: Check with a statistician whether paired Student's t-tests are correct. It is my understanding that when comparing cells that have undergone different treatments the test should be unpaired.

We thank the reviewer for this correction of our statistical analysis. We have now performed either unpaired t-tests or Mann-Whitney rank-sum test (for the comparison of two samples, one treatment/condition), one-way ANOVA or Kruskal-Wallis test (for the comparison of multiple samples, one treatment/condition) or two-way ANOVA (for the comparison of multiple samples, multiple treatment/conditions e.g. sodium arsenite-treated samples) depending on the assumed

nature of how the data set is distributed (parametric or non-parametric, respectively). Moreover, we apologize for the lack of detail provided regarding statistics in the first version of our manuscript. We have endeavored to be much more explicit in the revised version, both in the methods and in the figure legends, regarding which statistical test was performed on which data set.

Reviewer #3: My major concern with the study is the analysis of PB-regulated cytokine mRNAs presented in Figures 4 and 8. The genes tested include IL-6, CXCL8, COX-2, GM-CSF, and IL-1B. These genes are commonly associated with many inflammatory or IFN cascades. Their upregulation and PB-regulation are not definitively linked, and it is more likely that their upregulation is a consequence of IFN/immune signalling than of PB disassociation.

We apologize to the reviewer for the lack of clarity regarding how PB dynamics can regulate cytokine mRNA level. PB regulation of the ARE-containing transcripts that encode many inflammatory cytokines is still a novel concept that has only been reported by our group and a few others (Corcoran, 2012, Corcoran 2015, Robinson 2022, Castle, 2021, Kleer 2021, Vindry 2017, Blanco 2014, Tenekeci 2016). It is true, PB dynamics do not control transcription of cytokines, rather, PBs regulate some cytokine transcripts post-transcriptionally. RNA regulation in PBs provide a 'checkpoint' that controls the expression of potent inflammatory molecules. When an inflammatory stimulus (e.g. viral infection or TNF) induces the transcription of an ARE-containing RNA such as IL-6, this RNA is extremely labile and displays a very short half-life unless a second signal (e.g. activation of p38/MK2) induces PB disassembly, protecting the PB-regulated transcript from constitutive decay/repression in PBs. In summary, PBs provide a second layer of regulation for inflammatory molecule expression that is not fully appreciated. PBs also provide a means by which a cell can more rapidly translate inflammatory cytokines from RNA transcripts that are trapped in PBs and released when PB disassembly is induced by viral infection. In our manuscript, RNA FISH demonstrated that IL-6 and TNF RNA transcripts are found in PBs (Fig 9A). When PBs are lost due to expression of SARS-CoV-2 N protein, we showed that IL-6 and TNF RNA redistributed to the cytoplasm and the probe signal intensity increased (Fig 9B). This suggests that PBs regulate gene expression two ways: 1) increasing abundance of RNA transcripts that are normally subjected to rapid turnover in PBs and 2) redistributing these transcripts to the cytoplasm for translation. With our work, we do not mean to imply that the upregulation of cytokine mRNA during infection is solely because of PB disassembly, but that PB disassembly is one contributor (along with transcriptional upregulation) to the enhanced expression of inflammatory molecules who's cognate RNAs harbour AREs. In response to the reviewer's statement that "*it is more likely that their upregulation is a consequence of IFN/immune signalling than of PB disassociation*", we suggest that these mechanisms are not mutually exclusive, and it is more likely that both transcription and protection from RNA repression/decay in PBs contribute to inflammation.

Additionally, in the functional characterisation of these genes in N overexpression (Fig 8), there is no convincing link between the PB disassembly and expression of these genes. Indeed, the expression of these genes is not significantly associated with SARS-CoV-2 N protein overexpression as only one cytokine (TNF) was significantly increased. The title of Figure legend 8 "Ectopic expression of SARS-CoV-2 N elevates selected ARE-mRNAs" is therefore

misleading as there is only one upregulated mRNA that is statistically significant. Additionally, the data showing the no effect of TNF treatment on PB numbers should be presented.

We thank the reviewer for this astute assessment of our data. We have re-graphed the cytokine RNA analysis (in Fig 10 of the revised manuscript) to better represent the variance in our control samples. These RT-qPCR data fail to reach statistical significance (p=0.06 for TNF) and we have altered the title of the figure accordingly. That said, our analysis of PB-regulated cytokine RNAs by IF-FISH provides strong support that 1) TNF and IL-6 transcripts localize to PBs and 2) TNF and IL-6 transcripts redistribute to the cytoplasm and increase in abundance upon N protein expression and PB loss (Fig 9). With these two experiments in mind, we have reframed the major conclusions of our manuscript to consider the FISH data and limited making conclusions from RT-qPCR experiments. We also note that RT-qPCR is a population-based method that will not capture a change in re-localization of cytokine RNA transcripts from PBs to the cytoplasm that we observed by IF-FISH, and the lack of significance we observe by RT-qPCR does not translate to a lack of biological significance.

In response to this reviewer's comment, we performed new experiments and confirmed that TNF treatment of HUVECs did not significantly change PB numbers. We have added these data to a new supplemental Fig S8.

It is surprising that authors did not use their Luciferase-ARE reporter assay (included in the pre-print version) to directly link the effect of SARS-CoV-2 infection and protein expression on PB-associated turnover of ARE-containing mRNA transcripts.

We appreciate that the reviewer is familiar with our work and the ARE-containing luciferase reporter assay. We have chosen not to incorporate the assay into this manuscript, opting for the more biologically relevant analysis of ARE-containing cytokine transcripts that are elevated in severe COVID, especially IL-6 and TNF. Our work shows that these RNAs localize to PBs in basal conditions, and that their levels increase in response to ectopic expression of the N protein (Fig 9).

For Figure 6: While it looks like there is a difference in PB numbers/fold change between control (EV) and N in Figures 6 D and F, unfortunately, there are no statistics performed. I suppose this may be due to only two replicates of this experiment. At least 3 experiments are required to clarify this. Additionally, it is peculiar that there appears to be slightly more PB assembly in the sodium arsenate treatment when comparing the empty vector and N protein assembly. The reasons for the lack of effect on N on PB disassembly following sodium arsenite treatment need to be at least discussed.

We have repeated ectopic expression of N protein +/- sodium arsenite. We have added statistical analysis of these data to Fig 7 of the revised manuscript and added additional discussion of these data in the results section.

Fig 7. There seem to be lower expression for OC43 and NL63 N proteins than for SARS-CoV-2 which could partially explain lesser effect on PB disassembly. Could authors comment on this?

Some form of quantitative analysis of protein expression in western blots in this and other figures (e.g. densitometry of bands) should be presented to justify the conclusions.

We thank the reviewer for this astute observation. We do agree that different huCoV N proteins are not expressed equally, and we cannot fully discount expression level as the reason for the discrepancy in PB disassembly. We have performed densitometry of our immunoblots as requested by the reviewer (Fig S7); however, we note that the immunoblots were not all performed using the same antibody, making the direct comparison between signals impossible. A statement to reflect this has been added to the results section of the manuscript. However, we note that for all quantification of PB loss after N protein expression, our CellProfiler pipeline has been set to quantify PB counts only in cells with high expression of N protein. Therefore, although the population as a whole may have lower expression, in our PB analysis we considered only N protein-positive cells.

It is unclear how many experiments were undertaken and what data represents what biological samples for many of the figures.
The convention in the figure legends appears to be set out by the figure legend one for western blots. "Representative images from one of two independent experiments are shown. n=2." However, only the "n=x" is shown for figures with bar graphs but not pooled for the analysis or mentioned if the trends are the same. If possible, can the results from all experiments be pooled to show the robustness of the data and treatments?

We apologize for the confusion, and we have clarified our figure legends accordingly. We have also re-graphed all the PB data to represent PBs/cell for every cell counted in each independent experiment. These new graphs show each independent experiment separately so that the reviewer and reader can view the variability within and between experiments.

For figure 1, Hedls puncta are quantitated for figure 1 but not for Figure 2. Is there any reason why this was omitted for the other CoVs or can the authors defend the sole use of one puncta quantitation? This is important given that for figure 3, there is quite a pronounced reduction in DDX6 in whole-cell lysates, and while it would make sense there would be less PB machinery, is there the same reduction in Hedls puncta.

We are unable to co-stain OC43-infected cells for N protein and Hedls because both antibodies are mouse. Similarly, we are unable to co-stain 229E-infected cells with the J2 antibody and Hedls for the same reason. Therefore, we co-stained OC43- and 229E-infected cells with DDX6 and Hedls. We have added these new images and the quantification of Hedls puncta/cell to the new figure, Fig S3.

For figure 3 the title is too strong given that one of these viruses does reduce DDX6. For western blots used for comparison and quantitation, could densitometry analyses be performed and statistically assessed.

We thank the reviewer for this observation. We have altered the figure title to accurately reflect these data. We have added densitometry analysis of the immunoblots shown in revised Fig 4 and Fig 8; these analyses are found in new supplemental figures Fig S4 and Fig S7, respectively.

In figure 5D I am not sure what C145A corresponds to in the western blot, and it is not clear what it is from the text.

C145A is a mutant form of nsp5 that lacks catalytic activity. We were initially interested in testing this mutant in our screen because other viral proteases have been shown to cause PB disassembly in a protease-dependent manner. However, our data did not support an effect on PBs mediated by nsp5. For that reason, we have removed the C145A mutant from the analysis shown in Fig 6 of the revised manuscript.

Line 444: Origin of FBS.

We apologize for the oversight. FBS was purchased from Thermo Fisher. This information has now been added.

Line 467: I believe "polyethylimine" should be polyethylenimine

We apologize for our mistake; the reviewer is indeed correct and we have corrected the error in our text of the methods.

Section Lines 493: Could it be clarified what the target host transcript(s) is/are being used for normalisation.

We apologize for this oversight. We have added this information to the qPCR section in methods.

Line 505: As there are phenotypic differences between isolates and clades of SARS-CoV-2, could the GISAID accession number associated with this isolate be added to the text here? Also, maybe one comment about the lineage designation (Alpha Beta etc).

We apologize for this oversight and have added this information to the methods.

Line 532: Could the version of CellProfiler be added to this.

We apologize for neglecting this information. We used CellProfiler 4.0.6. This information has been added to the methods.

Line 594: G should be F

We apologize for our mistake and have corrected the figure labeling error.

References

Hubstenberger A, Courel M, Bénard M, Souquere S, Ernoult-Lange M, Chouaib R, et al. P-Body Purification Reveals the Condensation of Repressed mRNA Regulons. Mol Cell. Elsevier Inc; 2017;68: 144–157.e5. doi:10.1016/j.molcel.2017.09.003

Bakheet T, Hitti E, Khabar KSA. ARED-Plus: an updated and expanded database of AU-rich element-containing mRNAs and pre-mRNAs. Nucleic Acids Research. 2018;46: D218–D220. doi:10.1093/nar/gkx975

Blanco FF, Sanduja S, Deane NG, Blackshear PJ, Dixon DA. Transforming growth factor β regulates P-body formation through induction of the mRNA decay factor tristetraprolin. Mol Cell Biol. 2014;34: 180–195. doi:10.1128/MCB.01020-13

Corcoran JA, Khaperskyy DA, Johnston BP, King CA, Cyr DP, Olsthoorn AV, et al. Kaposi's sarcoma-associated herpesvirus G-protein-coupled receptor prevents AU-rich-element-mediated mRNA decay. J Virol. 2012;86: 8859–8871. doi:10.1128/JVI.00597-12

Corcoran JA, Johnston BP, McCormick C. Viral Activation of MK2-hsp27-p115RhoGEF-RhoA Signaling Axis Causes Cytoskeletal Rearrangements, P-body Disruption and ARE-mRNA Stabilization. Robertson ES, editor. PLoS Pathogens. Public Library of Science; 2015;11: e1004597. doi:10.1371/journal.ppat.1004597

Robinson, C., G. K. Singh, M. Kleer, E. L. Castle, B. Q. Boudreau and J. A. Corcoran. 2022. Kaposi’s sarcoma-associated herpesvirus (KSHV) utilizes the NDP52/CALCOCO2 selective autophagy receptor to disassemble processing bodies. *Plos Path*. Revised version resubmitted. https://doi.org/10.1101/2021.02.07.430164

Castle, E.L., C. Robinson, P. Douglas, K.D. Rinker, and J.A. Corcoran. 2021. Viral manipulation of mechanoresponsive signaling disassembles processing bodies. *Molecular and Cellular Biology.* 41(11):e0039921

Kleer, M., G. MacNeil, N. Adam, E.S. Pringle, and J.A. Corcoran. 2021. A panel of KSHV mutants in the polycistronic kaposin locus for precise analysis of individual protein products *J. Virol*. doi: 10.1128/JVI.01560-21

Franks TM, Lykke-Andersen J. TTP and BRF proteins nucleate processing body formation to silence mRNAs with AU-rich elements. Genes Dev. 2007;21: 719–735. doi:10.1101/gad.1494707

Vindry C, Marnef A, Broomhead H, Twyffels L, Ozgur S, Stoecklin G, et al. Dual RNA Processing Roles of Pat1b via Cytoplasmic Lsm1-7 and Nuclear Lsm2-8 Complexes. CellReports. 2017;20: 1187–1200. doi:10.1016/j.celrep.2017.06.091

Tenekeci U, Poppe M, Beuerlein K, Buro C, Müller H, Weiser H, et al. K63-Ubiquitylation and TRAF6 Pathways Regulate Mammalian P-Body Formation and mRNA Decapping. Mol Cell. 2016;62: 943–957. doi:10.1016/j.molcel.2016.05.017

Li Y, Chen R, Zhou Q, Xu Z, Li C, Wang S, et al. LSm14A is a processing body-associated sensor of viral nucleic acids that initiates cellular antiviral response in the early phase of viral infection. Proc Natl Acad Sci USA. 2012;109: 11770–11775. doi:10.1073/pnas.1203405109

Ayache J, Bénard M, Ernoult-Lange M, Minshall N, Standart N, Kress M, et al. P-body assembly requires DDX6 repression complexes rather than decay or Ataxin2/2L complexes. Matera AG,

editor. Mol Biol Cell. The American Society for Cell Biology; 2015;26: 2579–2595. doi:10.1091/mbc.E15-03-0136

Nancy Kedersha, Georg Stoecklin, Maranatha Ayodele, Patrick Yacono, Jens Lykke-Andersen, Marvin J. Fritzler, Donalyn Scheuner, Randal J. Kaufman, David E. Golan, and Paul Anderson. 2005. Stress granules and processing bodies are dynamically linked sites of mRNP remodeling. 169: 871–884.

Al-Souhibani et al. 2010 The RNA binding zinc finder protein tristetraprolin regulates AU-rich mRNAs involved in breast cancer related processes. Oncogene

Eri Seto, Reiko Yoshida-Sugitani, Toshihiko Kobayashi, Noriko Toyama-Sorimachi. 2015 The Assembly of EDC4 and Dcp1a into Processing Bodies Is Critical for the Translational Regulation of IL-6. PLoS One (5):e0123223. doi: 10.1371/journal.pone.0123223.
