# Supplementary material for: Human coronaviruses disassemble processing bodies
Source: PLoS Pathog. 2022 Aug 23;18(8):e1010724. doi: 10.1371/journal.ppat.1010724 (PMC9439236; doi:10.1371/journal.ppat.1010724)
Supplement: Attachment — Submitted filename: Corcoran Kleer Mulloy Response to reviewers FINAL.pdf [file ppat.1010724.s011.pdf]

Response to Reviewers

Major Comments

Reviewer #3: Authors provide new data (Fig 9B,C) that they claim are showing release of IL6 and TNF mRNAs from disassembled PBs in cells transfected with N-expressing plasmid along with qRT-PCR data of total cell RNA for these mRNAs. While I can accept that Fig 9A does show some co-localization of IL6 and TNF mRNAs in mock cells treated with TNFa, there is no FISH marker for PBs in Fig 9B (as it was in Fig 9A) to justify their conclusion. Yes, there appears to be more TNF mRNA in N-expressing cells but without PB marker it is not possible to conclude that this is the result of TNF mRNA release from disassembled PBs. RT-qPCR of total cell RNA while does show more TNF mRNA (only two repeats though) it doesn't prove that this mRNA is released from disassembled PBs.

I think authors should either provide definitive experimental proofs that cytokine mRNAs are indeed released from disassembled PBs in N-expressing cells or remove this conclusion from the manuscript.

This also refers to their argument against showing data on ARE-containing luciferase reporter assay as RT-qPCR data in the new Fig 9C show no statistically significant difference in IL6 and TNF mRNAs in N-expressing cells.

We thank the reviewer for this insightful suggestion. In response, we have performed a new experiment, now shown in Fig 9 panels D and E of the revised version of the manuscript. In this new experiment, HUVECs were transduced to express SARS-CoV-2 N, and IF-FISH was performed to stain for ARE-containing RNAs encoding TNF and IL-6 while also co-staining for PBs using antibodies for the PB-resident protein Hedls and the SARS-CoV-2 N protein. This new experiment demonstrated that in cells expressing the N protein, PB foci decrease (as shown consistently throughout the paper) and the PB loss in N protein-expressing cells is concomitant with an increase in the intensity of the RNA FISH signal for the TNF transcript (Fig 9 panel C). By co-staining for PBs and N protein in the same cells that were also stained for TNF RNA, we show that those cells which have greater staining intensity are also the ones which are i) expressing SARS-CoV-2 N protein and ii) devoid of PBs, thereby justifying our conclusion that PB-localized RNA encoding TNF re-localizes from PB foci to the cytoplasm in N protein-expressing cells.

Minor Comments

Reviewer #2: New figure 9C. it is unclear to me what the errorbars stand for and why there is no statistics. In the discussion you mention that differences in RNA levels (Fig10) are insignificant, though in your rebuttal and main text of the manuscript you do refer to IL6 and TNF as being upregulated and/or having a higher signal intensity. Without statistics I do think you have to carefully formulate this conclusion, especially regarding IL6 as differences both in figs 9 and 10 are small and have considerable variation between replicates. Perhaps doing statistics and reporting the p-values, even when not significant, can make these results better interpretable to readers.

We thank the reviewer for expressing this concern. As IF-FISH and RNA FISH were new methods in our lab, developed for the purpose of revising our first submission of this manuscript, we prioritized co-staining of ARE-containing RNAs with PB foci. After examination of FISH-stained samples with and without N protein expression, we observed the enhanced intensity of the cytoplasmic FISH signal using probes for TNF and IL-6 but were unable to complete three independent experiments in the time allocated for our first revision. We have now completed the third experiment, and these data have been added to Fig 9C. After statistical analysis was performed on measurements of cytoplasmic FISH signal intensity, we found that the signal intensity for the TNF RNA transcript was significantly elevated in the cytoplasm of N protein-expressing cells, whereas the intensity of the IL-6 RNA signal, though elevated upon N protein expression, was not statistically different from the control cells. We have revised our results and discussion to reflect this result.

Reviewer #3: Why there is now no PB disassembly in N-expressing cells vs EV control cells in Fig 7D? This contradicts other findings in this manuscript claiming key role of N protein in PB disassembly.

We thank the reviewer for pointing out that indeed, in Fig 7D of our prior submission, N protein expression did not cause significant PB disassembly after three independent experiments. However, we note that in our manuscript, there are a total of seven data panels, each representing separate sets of independent experiments wherein we expressed N protein and quantified PB loss. Of these, six data panels showed that N protein expression induced significant PB loss and one (in Fig 7D) did not show significant PB loss compared to controls. (The figures we are referring to are Figures 6D (HeLa system, n=4), 7A (n=3), 7E (n=4), 8B (n=3), 8D (n=3), 8F (n=3)). After the reviewer raised this point about our data in Fig 7D, we examined the PB counts closely, and note that the second experiment shown on our graph is clearly an outlier. In this outlier experiment, the untreated HUVEC population displayed very low PBs, making it difficult to show that PBs were lost because their starting level was so low. Since HUVECs are primary cells, we do observe variability of baseline PB counts between experiments and between batches of cells. Because of this, we reported PB counts per cell for every cell counted in each independent experiment (each dot on the graph represents PBs per cell counted), thereby providing the reader and reviewer the ability to view the data and assess data variability between our controls as well as the experimental condition. To specifically address the reviewer concerns, we performed the experiment shown in Fig 7D two additional times and these new data have been added to Fig 7D of the newly revised manuscript (which now contains n=5). We did not remove the outlying experiment, as it is an accurate reflection of the biological variability of this phenotype in primary cells; rather, we added the additional data onto existing analyses. Because of the outlying experiment, these data were still not deemed significant by our tests; however, we are confident that the reviewer and the editor will accept this honest representation of the phenotype, given that six of seven figures in our manuscript showed that N protein expression induces significant PB disassembly.
