# Supplementary material for: Human coronaviruses disassemble processing bodies
Source: PLoS Pathog. 2022 Aug 23;18(8):e1010724. doi: 10.1371/journal.ppat.1010724 (PMC9439236; doi:10.1371/journal.ppat.1010724)
Supplement: Attachment — Submitted filename: Kleer Mulloy Response to Reviewers.pdf [file ppat.1010724.s012.pdf]

**<u>Kleer Mulloy et al.</u>**
**<u>Response to Reviewers</u>**

## Comments

***Reviewer #2***

*The authors have sufficiently addressed my concerns by adding new experiments in what is now fig 9 and clarifying the methods and statistical analyses. I have no further comments.*

We would like to thank the reviewer for appreciating the improvements that we made to our prior manuscript.

## Major Issues

***Reviewer #3***

*<u>COMMENT 2</u>*
*While I appreciate that the authors have addressed our concerns with the follow up experiment (Fig 9D,E), to the untrained eye it does not look like there is much of an increase in signal in the bottom panel TNF RNA transcripts in the new Fig 9E (between the two EV and SARS-CoV-2 N bottom panels). I believe we should be seeing more signal or more diffuse RNA transcript signal? Could this be made clearer either through quantitation here in this figure or in a supplementary figure (there is white space under the image that could be used).*

*<u>COMMENT 1</u>*
*With the addition of this experiment and figure it appears that in SARS-CoV-2 N overexpressing cells there is a considerable increase in the Hedls protein level as compared to the EV (Fig 9D,E)? I understand that Hedls protein level was not changed in the SARS-CoV-2 infection at 6 or 12 hours (Fig 4A). Additionally, It doesn't look like the PBs (appearance of puncta) are reduced or disassembled. Instead it appears that the surrounding signal is stronger suggesting a higher abundance of Hedls rather than disassembly and cytoplasmic redistribution. Is this what was expected from this experiment? Could the authors clarify this observation?*

*<u>COMMENT 3</u>*
*In addition, I raised the concern about lack of demonstrated PB disassembly in Fig 7D, which the authors explained as an outlier experiment. However, new Fig 9D and E also in my opinion don't show PB disassembly (see comment above). Are these data also come from an outlier experiment? This needs to made clear in the text.*

## <u>Response to Major Issues</u>

In response to the reviewer's prior concerns, we added new data to our manuscript – these data were found in Fig 9D-E in the previous revised version but have been moved to Fig S9C-D in the most recently revised version of the paper. Reviewer 3 has expressed concern about these new

data with three separate comments. In the response that follows, we will address each of these comments separately in the order that they are numbered above.

***Comments by Reviewer 3 from prior review***: *"Authors provide new data (Fig 9B, C) that they claim are showing release of IL6 and TNF mRNAs from disassembled PBs in cells transfected with N-expressing plasmid along with qRT-PCR data of total cell RNA for these mRNAs. While I can accept that Fig 9A does show some co-localization of IL6 and TNF mRNAs in mock cells treated with TNFa, there is no FISH marker for PBs in Fig 9B (as it was in Fig 9A) to justify their conclusion. Yes, there appears to be more TNF mRNA in N-expressing cells but without PB marker it is not possible to conclude that this is the result of TNF mRNA release from disassembled PBs. RT-qPCR of total cell RNA while does show more TNF mRNA (only two repeats though) it doesn't prove that this mRNA is released from disassembled PBs."*

Response to COMMENT 1
From their prior appraisal of our manuscript (excerpted above), Reviewer 3 accepted that there was a greater amount of TNF RNA in N protein-expressing cells and requested that we perform IF-RNA FISH in cells that were co-stained for PBs to show TNF RNA signal increases in cells subject to PB loss. In the original RNA FISH experiment, these cells were co-stained for Hedls to label PBs and PB disassembly was verified qualitatively, but in earlier versions of this manuscript we did not quantify PB foci in these experiments because of our confidence that PBs disassemble following N expression as evidenced throughout the manuscript (Fig 6A, 7B, 7F, 8B, 8D, and 8E). Now, using the same slides that we used for RNA quantification experiments presented in Fig 9B-C, we have quantified PB loss in control versus SARS-CoV-2 N protein-expressing cells. These quantified data, along with representative images of PBs in control versus N protein-expressing cells, have been added to Fig S9A-B of the current version of the manuscript and show that SARS-CoV-2 N protein expression induced PB disassembly in the same experimental samples used to demonstrate that N protein enhanced TNF transcript abundance. These data add significantly to our conclusions and alleviate Reviewer 3's concern that PB disassembly did not occur in the experiments used to measure GAPDH, IL-6 and TNF RNA transcript intensity.

We note that in these IF-FISH experiments, two factors made PB foci more challenging to quantify than in the other experiments presented in the paper. First, in this experiment, Hedls/PB foci were labeled with an Alexa488-conjugated secondary antibody to minimize any spectral overlap with the RNA FISH probes (labeled with Quasar 670; see Figure 1 for spectra). Our CellProfiler quantitation method is optimized for staining PBs using Alexa555 as we have found that this fluorophore provides greater signal-noise ratio for PB foci regardless of the primary antibody used. Second, the IF-FISH protocol necessitated a second fixation step plus several hybridization and wash steps after the completion of the IF portion of the technique; in our hands, these additional manipulations decreased the signal-noise ratio in the PB channel. We have added additional information about imaging and quantification conditions to the methods and figure legends of the revised manuscript to increase transparency for the reviewer and reader.

Response to COMMENT 2

Ideally, the experiment shown in Fig 9B-C and Fig S9A-B would have permitted the simultaneous visualization of RNA transcripts, PBs, and N protein as previously requested by the reviewer. However, accurate quantification of PB loss is not possible in experiments with quadruple staining for PBs, RNA, nuclei (must be labeled as a reference point for CellProfiler) and N protein simultaneously because of both cross excitation and bleed through issues with the technically challenging experiment. We did indeed perform this experiment; these data were presented in Fig 9D-E of the prior submission and have been moved to Fig S9C-D of the current submission. To achieve this, we altered our staining protocol to label PBs with an Alexa 488-conjugated secondary antibody and N protein with an Alexa 555-conjugated secondary antibody. We did this to achieve zero spectral overlap between the PB fluorophore (Alexa 488) and the RNA FISH fluorophore (Quasar 670). We then used laser scanning confocal imaging to minimize cross-excitation and bleed through; yet despite these efforts, we were unable to perform any quantitation of these samples. The most significant challenge was caused by the overlap of emission spectra from Alexa 488 and Alexa 555 fluorophores (Figure 1 below), combined with the strong intensity of the staining for the SARS-CoV-2 N protein juxtaposed with the weaker intensity of the PB signal. This combination caused signal bleed through from the Alexa 555 (N protein) channel into the Alexa 488 (PBs) channel in N-expressing cells, despite attempts to minimize emission wavelength overlap. For this reason, it was not possible to quantify PB disassembly in the quadruple labeling experiment, as even by eye the images show increased brightness in the PB channel that is caused by N protein channel bleed through. We note that it is this N protein channel bleed though that Reviewer 3 has mistaken for increased PBs; clearly the effect is so pronounced that it would make quantification inaccurate. Due to spectral overlap concerns between Alexa555 and Quasar 670, we also chose not to quantify RNA signal intensity in the quadruple labeling experiment, deeming our prior quantitative analysis of RNA intensity (Fig 9B-C, n=3) when Quasar 670-conjugated RNA probes were co-stained only with Hedls/PBs labeled with Alexa 488 to prevent any possible spectral overlap as superior.

Nonetheless, the quadruple labeling experiment successfully connected prior observations of increased RNA signal intensity for the PB-regulated transcript TNF with N protein expression. It also qualitatively demonstrated that i) the intensity of the GAPDH RNA FISH signal was equal or greater in vector control cells (that possess PBs) than in N protein-expressing cells (that are devoid of PBs), confirming the quantified data presented for RNA FISH (Fig 9B-C) and PBs (Fig S9A-B) and reinforcing that GAPDH is not a PB-regulated RNA transcript and is unaffected by altered PB dynamics and ii) the intensity of TNF RNA FISH signal was increased in N protein-expressing cells compared to vector control cells, confirming data quantified in Fig 9B-C. This experiment compliments Fig 9B-C and improves the manuscript.

We have also provided additional images of this experiment in Fig S9. Three independent images of each condition are shown, and each of the channels are displayed separately so that the reviewer can visualize that the increased intensity in the Hedls/Alexa 488 channel is derived from the intense N protein staining in the Alexa 555 channel. In addition, we have inverted the RNA FISH channel to make it easier to discern the difference in RNA probe intensity between conditions. Taken together, we hope that the additional information and alternative presentation of the data will assuage the reviewer's concerns on this point and reinforce that the experiments presented in Fig S9 are not outlier experiments.

Response to COMMENT 3
We would like to note for Reviewer 3 that the outlier experiment shown in Fig 7 where N protein expression did not induce significant PB disassembly relative to control cells is an outlier because the level of PBs was too low in the control cells, not because PBs remained in N-expressing cells (see graph Fig 7D where we have plotted PB counts for every cell in every independent experiment to make this information visible to the reader and reviewer). Additional explanation of the reason for this outlier has been added to the text interpretation of Fig 7.

## Minor Issues

### *Reviewer #3*

*A minor issue to address is Dapi should be written DAPI in this figure and others and in multiple places in text.*

We apologize for this error and have altered the figure labels and the text accordingly.

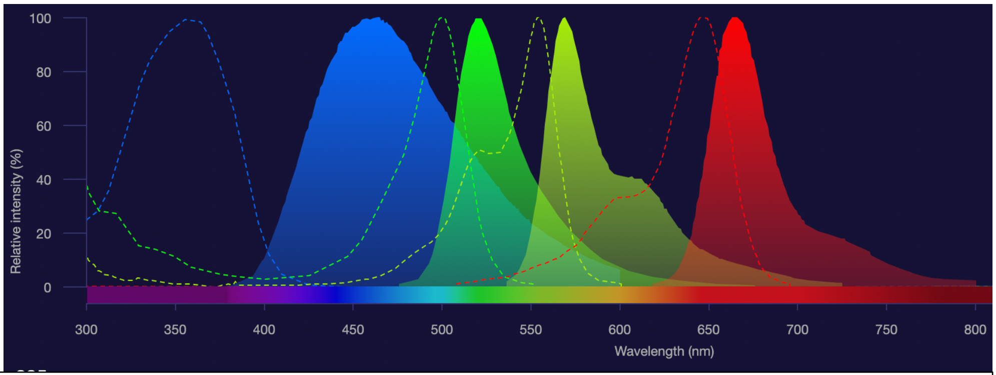


**Figure 1.** Spectra of the excitation (dotted lines) and emission (solid lines) wavelengths for the four fluorescent labels used in Fig S9. Blue = DAPI (nuclei); green = Alexa 488 (Hedls/PBs); yellow = Alexa 555 (N protein); red = Quasar 670 (RNA FISH). Taken from Fluorescence Spectra Viewer, Thermo Fisher Scientific.
